# Dicavitary twin pregnancy in patient with bicornuate bicollis uterine anomaly

**DOI:** 10.1002/ccr3.7440

**Published:** 2023-06-07

**Authors:** Mihiri Karunaratne, Dora J. Melber, H. Irene Su, Gladys A. Ramos

**Affiliations:** ^1^ University of California, San Diego School of Medicine La Jolla California USA; ^2^ Department of Obstetrics, Gynecology and Reproductive Sciences University of California, San Diego Health Sciences La Jolla California USA; ^3^ Division of Reproductive Endocrinology and Infertility, Department of Obstetrics, Gynecology and Reproductive Sciences University of California, San Diego Health Sciences La Jolla California USA; ^4^ Division of Maternal Fetal Medicine, Department of Obstetrics, Gynecology and Reproductive Sciences University of California, San Diego Health Sciences La Jolla California USA

**Keywords:** bicornuate uterus, pregnancy, twin, uterine didelphys, uterine duplication anomalies

## Abstract

**Key Clinical Message:**

Twin pregnancies in uterine didelphys and uterus bicornuate bicollis represent dicavitary twin pregnancies that can be managed using similar principles. Consideration must be given to delivery planning including mode of delivery and uterine incision.

**Abstract:**

Dicavitary twin pregnancies present unique challenges for obstetric management. This case demonstrates an approach to management of a bicornuate bicollis twin pregnancy and provides a contemporary review of the literature on dicavitary twin pregnancies.

## Graphical abstract

This case demonstrates an approach to management of a bicornuate bicollis twin pregnancy and provides a contemporary review of the literature on dicavitary twin pregnancies with a discussion of unique challenges for their obstetric management.

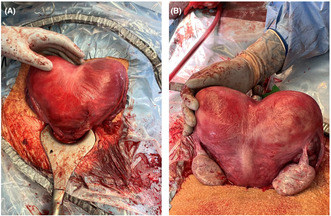

## 1 INTRODUCTION

The calculated prevalence of Mullerian anomalies is estimated to be about 5% while the prevalence may be higher in women with infertility, about 8%.^1^ The classification of Mullerian anomalies is problematic and there is no universally accepted classification. The American Society for Reproductive Medicine classification is the standard in the United States. Generally, the diagnosis of Mullerian anomalies relies on imaging including hysterosalpingography, 2‐D and 3‐D ultrasound, diagnostic hysteroscopy, magnetic resonance imaging, and rarely combined laparoscopy and hysteroscopy. A dicavitary uterus refers to two separate uterine cavities, but the diagnosis of the specific uterine anomaly can be challenging antenatally. Uterine bicornuate bicollis is a result of incomplete fusion of the Mullerian or paramesonephric ducts and is characterized by double or single vagina, double cervices and two single‐horned uteruses which show partial fusing of their muscular walls.^2^ Uterine bicornuate bicollis may be associated with renal anomalies as well as vaginal septum. Although rare, uterine bicornuate bicollis has been associated with higher rates of adverse pregnancy outcomes including miscarriage, fetal growth restriction, preterm delivery, malpresentation, and higher rates of cesarean delivery.^2^ Uterine didelphys, in comparison, is caused by a complete lack of fusion of the Mullerian ducts and characterized by two uterine cavities and two cervices with a longitudinal vaginal septum present in the majority of patients.^3^ It can be difficult to differentiate uterine bicornuate bicollis from uterine didelphys, as such, the former is often referred to as pseudodidelphys. In this case report we discuss a patient with a unique presentation of dichorionic diamniotic twin pregnancy with a twin in each horn of a bicornuate bicollis uterus. We also present a literature review of contemporary cases and outline the obstetric management and outcomes of patients with dicavitary pregnancies.

## 2 CASE HISTORY AND OUTCOMES

**FIGURE 1:**
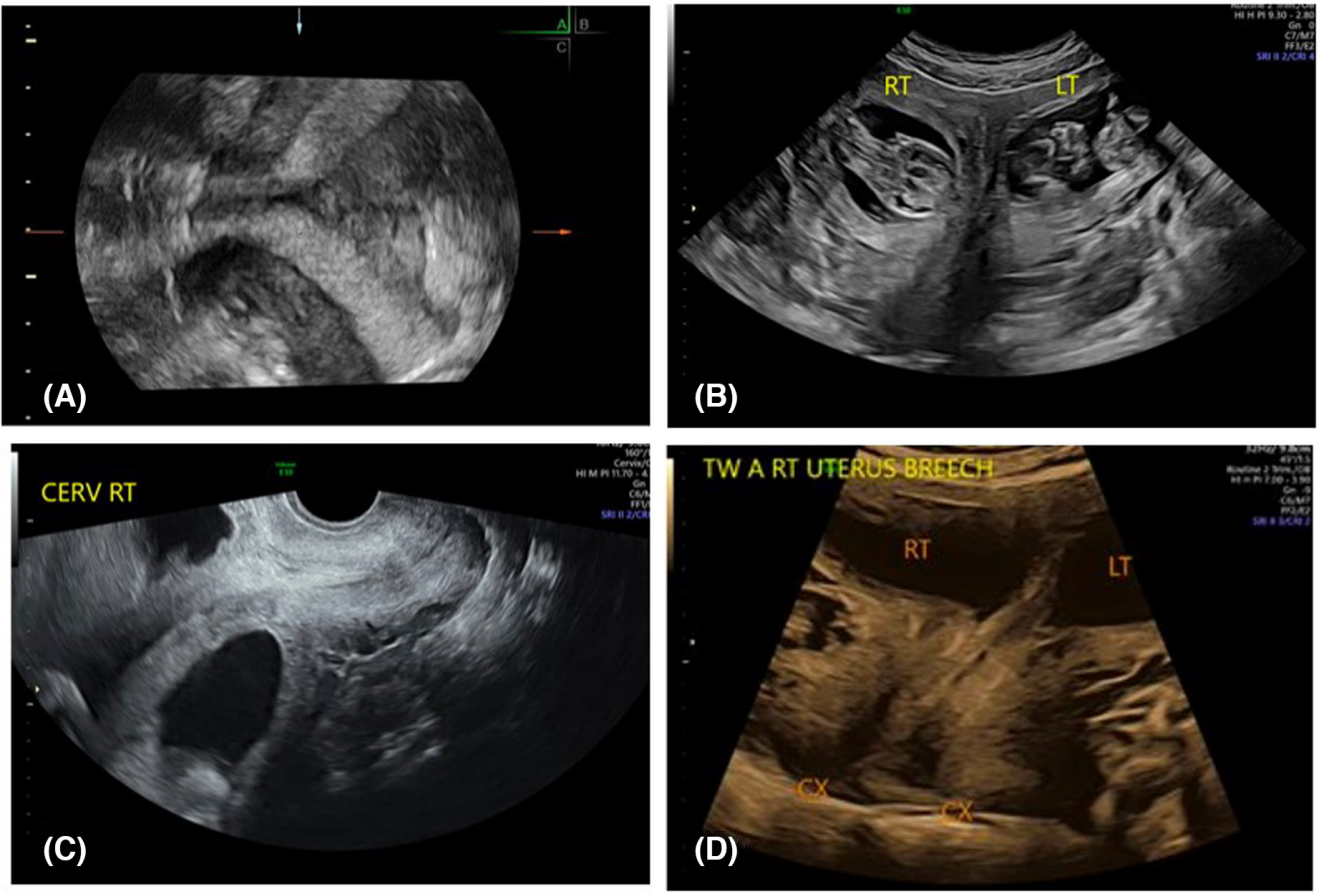
(A) Prepregnancy, transvaginal ultrasound with 3D reconstruction, (B) 12 weeks 2 days, transabdominal ultrasound, transverse image demonstrating two uterine cavities, (C) 12 weeks 2 days, transvaginal ultrasound, right cervix clearly visualized, (D) 18 weeks 2 days, transabdominal ultrasound, two uterine cavities with corresponding left and right cervices.

A 25‐year‐old woman with a diagnosis of polycystic ovarian syndrome was found to have a dicavitary uterine anomaly, initially thought to be uterine didelphys, discovered during a work‐up for infertility. A hysterosalpingogram performed at an outside clinic showed a solitary uterine horn extending to the right of midline with rapid spillage from the right fallopian tube, without evidence of a second uterine horn. However, on follow‐up transabdominal and transvaginal ultrasound two cavities were confirmed **(**Figure 1A **)**. Pelvic exam at this time revealed one normal‐appearing cervix to the right of a longitudinal vagina septum, but a second cervix was not palpable. A renal ultrasound showed bilateral, normal kidneys. She was treated with letrozole ovulation induction, and after one cycle, transvaginal ultrasound confirmed a viable pregnancy in each uterine horn. Due to the increased complexity of dicavitary twin pregnancies, her care was transferred to our Maternal‐Fetal Medicine Department.

At 12 weeks and 2 days, three‐dimensional ultrasound with multiplanar reconstruction showed a right uterine horn in communication with the vagina via a normal‐appearing cervix and a left uterine horn with the suggestion of a rudimentary cervical canal (Figure 1B,C). A second cervix or any communication between the right and left uterine horns could not be confirmed on this study. A subsequent endovaginal ultrasound at 16 weeks and 2 days confirmed the presence of both right and left cervix while a clear connection between the left cervix and the vagina was not appreciated. Anatomy survey at 18 weeks and 2 days revealed no congenital anomalies and normal cervical lengths **(**Figure 1D **)**. Monthly follow‐up growth ultrasounds confirmed normal interval growth of both fetuses.

**FIGURE 2:**
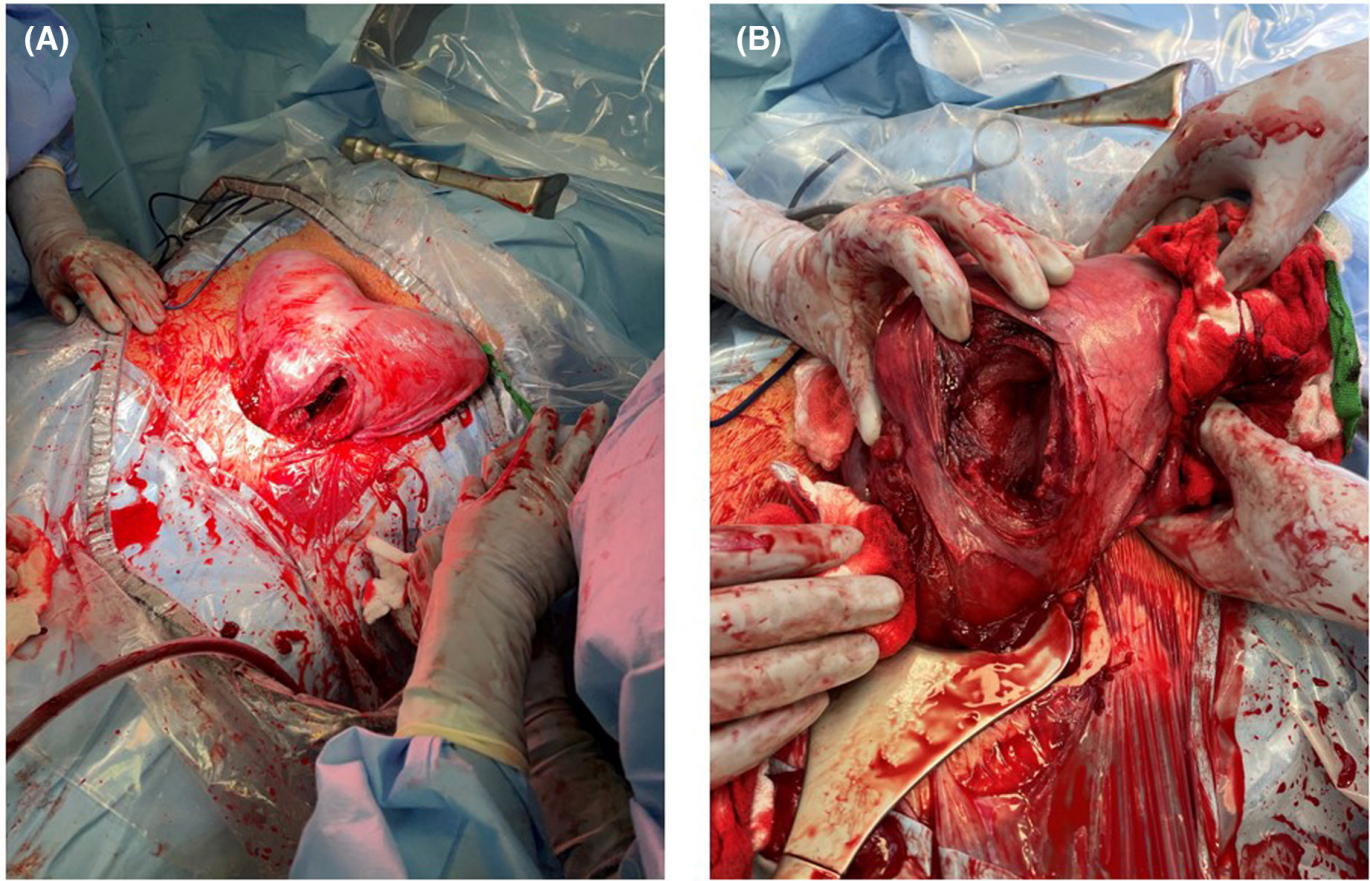
(A) Bilateral classical incisions were made to facilitate delivery. (B) Full‐length uterine septum was confirmed without communication between the two uterine cavities.

**FIGURE 3:**
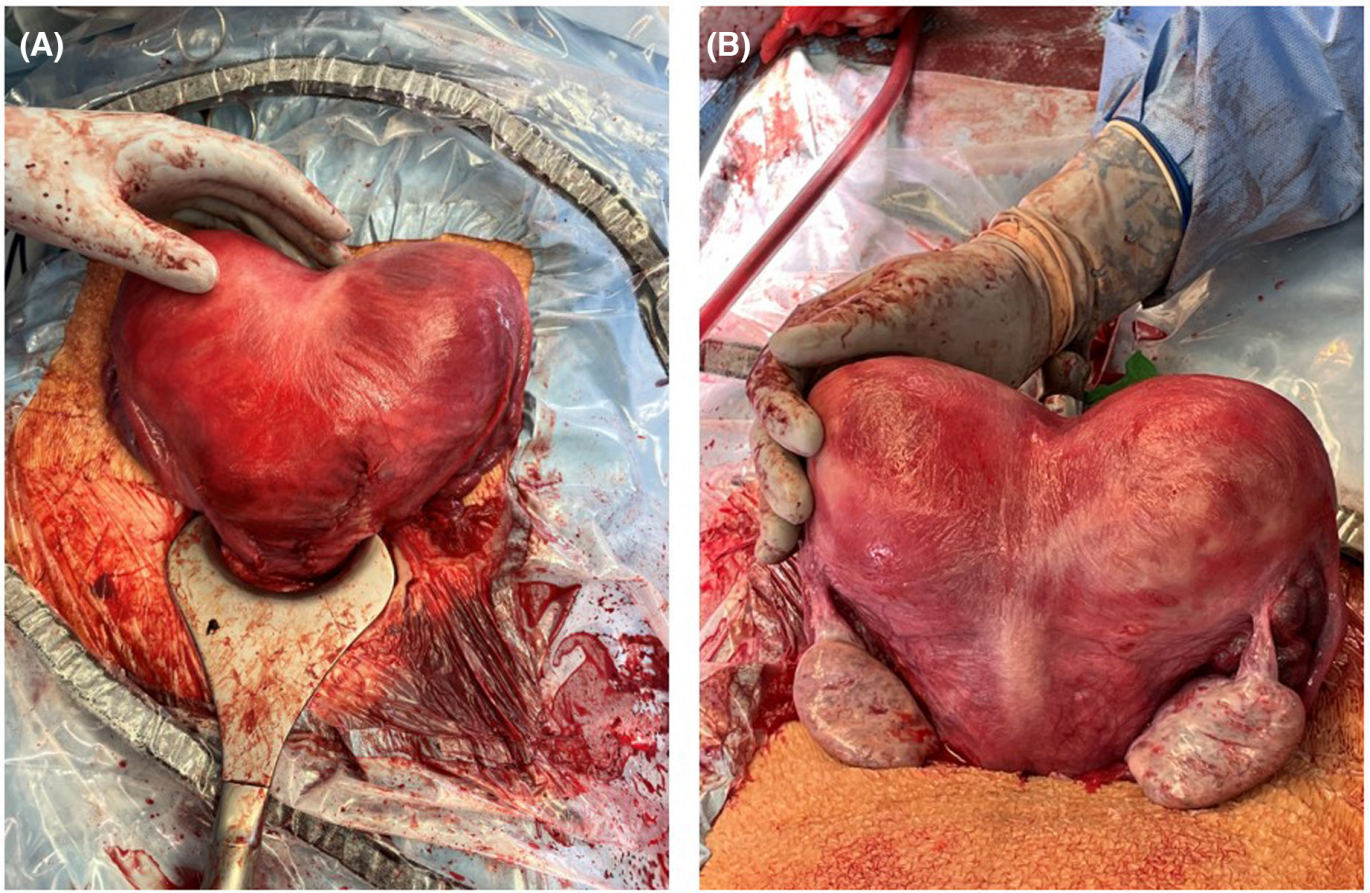
(A) Anterior view of the uterus after delivery and hysterotomy repair. (B) Posterior view of the uterus after delivery.

Although a second cervix had been identified, prior imaging and physical exam were unable to confirm definitive communication between the left uterine cavity and the vagina. Further, both twins were found to be malpresenting at term in the breech presentation, so the patient was scheduled for primary cesarean section at 37 weeks and 0 days. Exam under anesthesia confirmed a longitudinal vaginal septum and, for the first time, two cervices were palpated. Primary Cesarean section was performed under combined spinal‐epidural anesthesia via Pfannenstiel incision. Upon entry the uterus appeared heart shaped with >1 cm serosal indentation in the midline. This finding is most consistent with the American Society for Reproductive Medicine (ASRM) 2021 classification of uterus bicornuate bicollis.^4^ Fusion of the lower uterine segments precluded adequate independent assessment of the lower uterine segments; thus, two classical uterine incisions were performed. After delivery, a full‐length uterine septum was confirmed without communication between the two uterine cavities (Figures 2 and 3). The surgery was uncomplicated with total estimated blood loss of 1260 mL. Twin A was a 3350 g live male with Apgar scores of 8 and 8 who remained with mother after delivery. Twin B was a 2555 g live male with Apgar scores of 8, 7, and 8 who was transported to the NICU following delivery. The patient had an uneventful postpartum course. She was counseled regarding future considerations for her care to include PAP smears of both cervices and avoidance of laboring in future pregnancies given bilateral classical uterine incisions.

## 3 DISCUSSION

### 3.1 Classification

This report describes a rare case of dicavitary dichorionic diamniotic twin pregnancy. In this case, the optimal classification of the uterine anomaly is a uterus bicornuate bicollis with longitudinal septum.^4^ In review of the literature this anomaly may be misidentified as uterine didelphys, as was the case with our patient. In both, two separate uterine cavities are present. However, in the uterus bicornuate bicollis the lower uterine segments are fused externally in the midline leaving a deep indentation at the fundus producing a heart‐shape when viewed externally. Functionally, these anomalies are similar in that the two uterine cavities are separate from each other, with two distinct birthing canals made up of two cervices and often with a longitudinal septum present.

A review of published literature revealed 23 case reports describing dicavitary twin gestations in patients with dicavitary uterine anomalies since 1980. PubMed was searched using terms: uterine anomaly; Mullerian anomaly; uterine didelphys; uterine bicornuate bicollis; dicavitary gestation; dicavitary pregnancy; twin gestation; and twin pregnancy. We excluded cases of dicavitary anomalies with twin gestation in a single horn. Of the 23 contemporary cases, at least five are described as having features consistent with a diagnosis of uterus bicornuate bicollis by the ASRM classification^4^ (see Table 1). The prognosis, risks, and management strategies of dicavitary twin gestation are limited to a small number of case reports in the literature. We reviewed the literature to discuss several learning points relevant to this case: feasibility of vaginal delivery versus cesarean, surgical approach to cesarean, and delivery timing.

**TABLE 1:** Literature review of contemporary case reports of dicavitary twin gestations.

Case report, year	Age	Parity	Conception	EGA at delivery	Interval between deliveries	First twin delivery	Second twin delivery	Complications	Anatomy
Leiberman et al., 1980^5^	30	P6	Spontaneous	37w0d	< 1 day	Vaginal delivery—at home	Cesarean (low transverse)—failure to progress	None	*Uterus didelphys*: two uteri and two cervices
Nhân et al., 1983^6^	26	G3P2	Unknown	37w0d	< 1 day	Vaginal delivery	Vaginal delivery	None	*Uterus didelphys*: two uteri, two cervices, and vaginal septum
Kekkonen et al., 1991^7^	25	G2P1	Spontaneous	37w4d	0 days	Cesarean (low transverse)—due to malpresentation, following spontaneous labor	Cesarean (low transverse)—due to malpresentation, following spontaneous labor	Blood loss 1700 mL, 2 units PRBCs given	*Uterus didelphys*: two uteri, two cervices, and vaginal septum
Vandermolen et al., 1993^8^	29	G1P0	Ovulation induction	36w5d	0 days	Cesarean (low transverse)—due to malpresentation	Cesarean (low transverse)—due to malpresentation	Fetal growth restriction (×2)	*Uterus bicornuate bicollis*: two uterine cavities, two cervices, and vaginal septum
Brown et al., 1999^9^	34	G6P6	Spontaneous	26w0d	0 days	Cesarean (unspecified incisions)—emergent due to decelerations and breech presentation	Cesarean (unspecified incisions)—emergent due to decelerations and breech presentation	Placental abruption, preterm delivery	*Uterus didelphys*: two uteri, two cervices, and vaginal septum
Ahmad et al., 2000^10^	Unknown	Unknown	Spontaneous	31w0d	0 days	Cesarean (unspecified incisions)—due to chorioamnionitis and arrest of dilation following induction of labor	Cesarean (unspecified incisions)—due to chorioamnionitis and arrest of dilation following induction of labor	Fetal growth restriction, chorioamnionitis, arrest of dilation, preeclampsia	*Uterus didelphys*
Tyagi et al., 2001^11^	30	G3P2	Spontaneous	33w0d 33w5d	5 days	Vaginal delivery	Vaginal delivery—breech delivery following spontaneous labor	Preterm labor	*Uterus didelphys*: two uteri, two cervices, and vaginal septum
Singhal et al., 2003^12^	20	G1P0	Spontaneous	35w0d	0 days	Cesarean (low transverse)—due to malpresentation following preterm labor	Cesarean (low transverse)—nonviable	Fetal demise at 32 weeks of one twin, malpresentation of viable twin, preterm labor	*Pseudodidelphys*: two uterine cavities, two cervices (one hypoplastic), and vaginal septum
Nohara et al., 2003^13^	29	G2P0	Ovulation induction and bilateral IUI	25w0d 35w0d	66 days	Vaginal delivery (right horn)—preterm labor	Cesarean (low transverse)—due to fetal distress and minimal contraction in left horn in the setting of PPROM and preterm labor	PPROM, fetal distress at 25 weeks	*Uterus bicornuate bicollis*: two uterine horns, two cervices, and vaginal septum
Demaria et al., 2005^14^	23	G3P2	Unknown	27w0d	0 days	Cesarean (low transverse)—due to presumed placental abruption	Cesarean (low transverse)—due to presumed placental abruption	Right hemiuterus torsion diagnosed intraoperatively	*Uterus didelphys*: two uteri and two cervices
Allegrezza, 2007^15^	Mid 20s	G5P1	Spontaneous	31w0d	0 days	Vaginal delivery	Vaginal delivery	PPROM of both gestations	*Uterus didelphys*: two uteri, two cervices, and vaginal septum
Garg et al., 2010^16^	24	G1P0	Spontaneous	37w0d	0 days	Cesarean (low transverse)—scheduled	Cesarean (low transverse)—scheduled	None	*Uterus didelphys*: two uteri, two cervices, and vaginal septum
Jan et al., 2013^17^	26	G4P3	Spontaneous	35w2d 38w2d	23 days	Vaginal delivery—following PPROM	Vaginal delivery—following induction of labor	PPROM at 35w2d	*Uterus didelphys*: two uteri, two cervices, and vaginal septum
Maki et al., 2014^18^	32	G2P1	Ovulation induction	37w6d	0 days	Vaginal delivery	Cesarean (unspecified incisions)—due to recurrent late decelerations	None	*Uterus didelphys*: two uteri, two cervices, and vaginal septum
Yang et al., 2015^19^	37	G1P0	IVF and embryo transfer into each horn	39w0d	0 days	Cesarean (low transverse)—scheduled	Cesarean (low transverse)—scheduled	Postpartum hemorrhage with blood loss of 2200 mL and transfusion required	*Uterus didelphys*: two uteri, two cervices, and vaginal septum
Levy et al., 2015^20^	26	G2P0	Spontaneous	29w6d 32w1d	16 days	Vaginal delivery—following PPROM at 29w6d	Vaginal delivery—following PPROM at 30w3d and preterm labor	PPROM, postpartum hemorrhage with blood loss of 1550 mL, 4 units PRBCs and uterine tamponade balloon (right); PPROM and preterm labor (left)	*Uterus bicornuate bicollis*: two uterine horns, two cervices, vaginal septum, and previously resected imperforate obstructed right hemivagina
Li et al., 2016^21^	25	G3P1	Spontaneous	37w4d	0 days	Cesarean (low transverse)—following spontaneous labor and due to vaginal septum obstructing cervix	Cesarean (low transverse)—following spontaneous labor and due to vaginal septum obstructing cervix	None	*Uterus bicornuate bicollis*: two uterine cavities, two cervices, and vaginal septum; described as “bicorporeal septate uterus” with midline septum from fundus to cervices
Al Yaqoubi et al., 2017^22^	30	G4P1	Spontaneous	34w3d	0 days	Vaginal delivery	Vaginal delivery	Preterm labor at 34w	*Uterus didelphys*: two uteri, two cervices, and vaginal septum
Ani et al., 2018^23^	Unknown	G11P3	Spontaneous	Approximately 30w	0 days	Vaginal delivery—at home	Cesarean (unspecified incision)—due to retained demised second twin	Fetal demise, postpartum hemorrhage	*Uterus didelphys*: two uteri and two cervices
Post et al., 2019^24^	35	G1P0	Intra‐uterine insemination of one cervix	38w6d	0 days	Cesarean (low transverse)—scheduled due to fetal growth restriction and suspected hypoplastic left cervix	Cesarean (low transverse)—scheduled due to fetal growth restriction and suspected hypoplastic left cervix	Fetal growth restriction, postpartum hemorrhage with blood loss of 1.6 L and no transfusion given	*Uterus didelphys*: two uteri and two cervices (hypoplastic left cervix confirmed to be blind pouch intraoperatively); small communication between cervices on multiplanar US; ESHRE/ESGE U3b‐C3‐V0^25^
King et al., 2020^26^	27	G3P1	Ovulation induction	31w0d 31w1d	1 day	Vaginal delivery—following PPROM	Cesarean (low transverse)—emergent due to non‐reassuring fetal status and chorioamnionitis	PPROM at 29w5d, placenta left in situ (right uterus), chorioamnionitis (left uterus)	*Uterus didelphys*: two uteri, two cervices, and previously resected vaginal septum
Goulios et al., 2020^27^	35	Multiparous	Spontaneous	36w3d	0 days	Cesarean (low transverse)—due to malpresentation	Cesarean (classical incision)—due to malpresentation	Fetal growth restriction, preeclampsia	*Uterus didelphys*: two uteri, two cervices, and vaginal septum
Mohamad et al., 2020^28^	36	G6P3	Spontaneous	25w3d 35w3d	70 days	Cesarean (corporeal incision)—due to malpresentation and chorioamnionitis	Vaginal delivery—following trial of labor after cesarean	PPROM at 17w and chorioamnionitis at 25w (left), PPROM at 35w (right)	*Uterus didelphys*: two uteri and two cervices; ESHRE/ESGE U3b‐C2‐V0^25^
Our case, 2020	25	G1P0	Ovulation induction	37w0d	0 days	Cesarean (classical incision)—due to breech presentation and unconfirmed cervical connection	Cesarean (classical incision)—due to breech presentation and unconfirmed cervical connection	None	*Uterus bicornuate bicollis*: two uterine horns, two cervices, and vaginal septum

Abbreviations: EGA, estimated gestational age; IVF, in vitro fertilization; PRBCs, packed red blood cells; PPROM, preterm premature rupture of membranes.

### 3.2 Complications

Twin pregnancies with any concomitant uterine anomaly are at increased risk of requiring a cerclage, undergoing preterm birth, lower birth weights, and malpresentation at the time of delivery.^29^ A retrospective cohort of 49 cases of uterus didelphys in pregnancy found that 18% had obstructed hemivagina, 24% were delivered prematurely, 11% were complicated by growth restriction, with a cesarean delivery rate of 84%.^3^ Given the known increase in complications, any pregnancy with dicavitary twin pregnancy should undergo close monitoring, with consideration of referral to a tertiary care center.

### 3.3 Mode of delivery

Successful delivery via both vaginal route and cesarean section have been described. A higher risk of cesarean is to be expected given the increased risk of malpresentation and labor dystocia. However, a dicavitary gestation is not itself a contraindication for vaginal birth unless the birth canal is incomplete or obstructed. The most common indications for cesarean section in the literature include malpresentation,^7^, ^8^, ^9^, ^12^, ^27^, ^28^ fetal distress,^13^, ^18^, ^26^ labor dystocia,^5^, ^10^ and concern for obstruction of the birth canal.^21^, ^24^

In the case of our patient, a second cervix was never palpated or visualized on exam during the antepartum period, despite visualization of a second cervical canal on ultrasound. Given this concern and breech presentation of both twins at term, a cesarean was scheduled for 37 weeks gestation.

The presence of a pregnancy in both horns does not necessarily imply a communication between each uterus to the vagina. In fact, small connections between cervices have been described that may allow for fertilization of both uteri via one functional cervix.^24^ In our patient, an MRI prior to or during pregnancy may have helped to delineate the anatomy. However, given the fetal presentation, it would not have altered clinical management.

### 3.4 Incision

The decision to undergo a vertical (classical) incision versus a low‐transverse approach in any cesarean delivery is typically dependent on the evaluation of the lower uterine segment. In the case of a uterus bicornuate bicollis, the lower uterine segments are fused externally, limiting adequate evaluation. A low‐transverse incision risks extension, injury of the septum, difficult repair, hemorrhage, and may have an impact on future pregnancies. For these reasons, bilateral classical incisions were performed in this case. However, bilateral low transverse incisions have been successfully described in the literature.^7^, ^12^, ^14^, ^16^, ^19^, ^21^, ^24^ Goulios et al^27^ describe a case where LTCS was performed on the left, and a classical incision was deemed necessary on the right.

In the absence of contraindication to vaginal delivery, successful vaginal delivery for both twins have been reported.^6^, ^11^, ^15^, ^17^, ^20^, ^22^ In rare cases, a cesarean may be required for delivery of one twin, while the other is able to be delivered via the vaginal route, and vice versa.^5^, ^13^, ^18^, ^23^, ^26^, ^28^

### 3.5 Interval deliveries

It has previously been described that the two uteri of a dicavitary twin pregnancy can have independent functions of labor.

Maki et al.^18^ describes successful vaginal delivery followed by cesarean for the second twin due to fetal distress in the setting of uterine didelphys. During simultaneous labor, the timing of contractions were recorded for each uterus. The uterine horns contracted synchronously (within 5 s of each other) only 10% of the time.^18^

This phenomenon is further illustrated in cases of delayed‐interval delivery. Nohara et al.^13^ describes a case of uterus bicornuate bicollis in which a cesarean was performed at 25 weeks for fetal distress in the setting of PPROM and preterm labor. During the labor of the left uterine horn, the right side exhibited minimal contractions. After successful delivery, an interval of 66 days elapsed before preterm labor commenced in the opposite horn followed by vaginal delivery of the second twin at 35 weeks.^13^ Based on this case, the independent functioning of the two cavities does not appear to be precluded by external attachment.

Mohamad et al.^28^ describes a similar case of uterine didelphys where PPROM of one horn resulted in chorioamnionitis. After evacuation of the infected hemi‐uterus via cesarean section, a 70‐day interval elapsed before PPROM and successful vaginal birth after cesarean (VBAC) occurred at 35 weeks on the opposite side.^28^

### 3.6 Trial of labor after cesarean (TOLAC)

The risk of uterine rupture in the setting of a TOLAC in cases of dicavitary uterus is unknown. Successful VBAC has been described in cases of singleton pregnancies where the subsequent pregnancy presents in the opposite cavity^30^ or in the same uterine cavity.^31^ In cases of twin pregnancies cesarean of the first followed by vaginal delivery of the second twin during the same pregnancy has also been described.^13^, ^28^

Given limited data or consensus, delivery planning in cases of dicavitary pregnancies should be individualized. The care of patients with these pregnancies should include a multidisciplinary approach involving specialists in reproductive endocrinology and infertility, maternal fetal medicine, and neonatology. Given their complexity, management is best provided in a tertiary care setting.

## AUTHOR CONTRIBUTIONS

**Mihiri Karunaratne:** Data curation; methodology; visualization; writing – original draft; writing – review and editing. **Dora J. Melber:** Conceptualization; data curation; methodology; visualization; writing – original draft; writing – review and editing. **H. Irene Su:** Methodology; supervision; validation. **Gladys A. Ramos:** Conceptualization; methodology; supervision; validation; visualization; writing – original draft; writing – review and editing.

## FUNDING INFORMATION

None.

## CONFLICT OF INTEREST STATEMENT

The authors deny any conflict of interest.

## CONSENT

Written informed consent was obtained from the patient to publish this report in accordance with the journal's patient consent policy.

## Data Availability

Data sharing not applicable to this article as no datasets were generated or analyzed during the current study.
